# Supplementary material for: Microwave-free nuclear magnetic resonance at molecular scales
Source: Nat Commun. 2017 Jul 3;8:15950. doi: 10.1038/ncomms15950 (PMC5500877; doi:10.1038/ncomms15950)
Supplement: Supplementary Information [file ncomms15950-s1.pdf]

Type of file: PDF
Size of file: 0 KB
Title of file for HTML: Supplementary Information
Description: Supplementary Figures, Supplementary Table, Supplementary Notes and Supplementary References

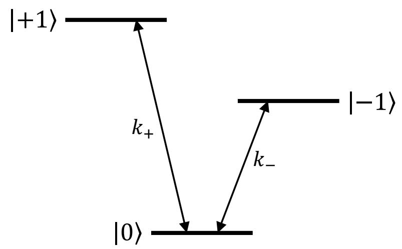

Supplementary Figure 1. **Model of the NV centre energy levels used for the rate equation derivation.** Model used to describe the population dynamics within the NV electronic ground state between the spin states $|0\rangle$, $|+1\rangle$ and $|-1\rangle$. The rate of relaxation to the $|-1\rangle$ state is increased by the on-resonance external proton signal.

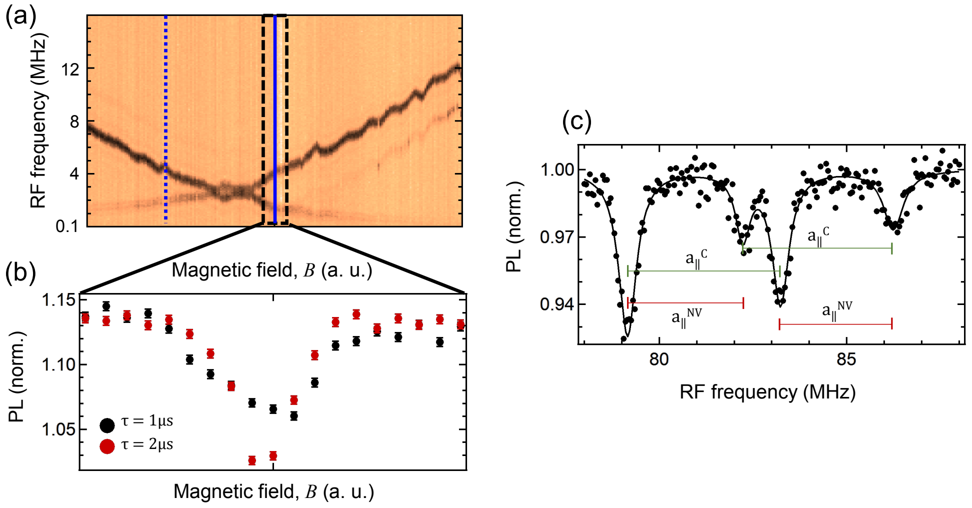

Supplementary Figure 2. **Motivation for sensing with ^{13}C coupled ^{15}NV centre.** (a) Optically detected magnetic resonance (ODMR) map across the GSLAC for a ^{15}NV. The NV-proton resonances are shown with the blue lines. (b) The signal zoomed near the NV-proton resonance past the GSLAC. This shows a far stronger decay than expected for an NV interacting with the proton bath. We put this down to the NV-proton crossing feature as explained in Ref. [1]. (c) ODMR taken for the NV with data presented in main text Fig. 3. This is a ^{15}NV with a strong coupling of $\approx$ 4 MHz to a ^{13}C causing a shift in the NV crossing feature away from the NV-proton resonance. Error bars correspond to one standard deviation.

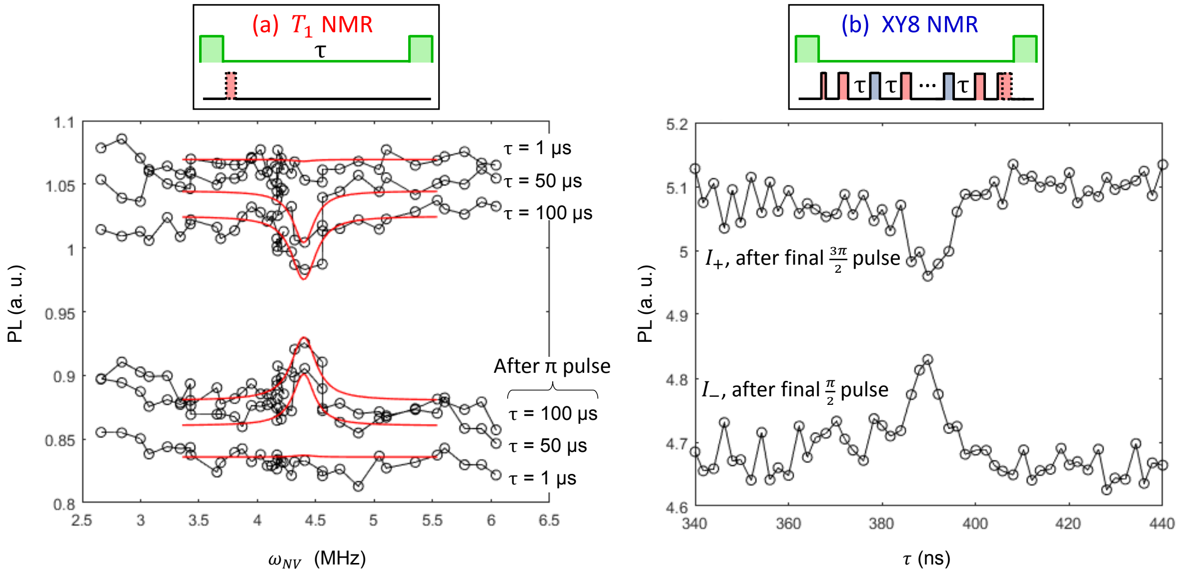

Supplementary Figure 3. **Data for comparison of $\mathbf{T_1}$ and XY8 techniques.** (a) T_1 spectra near the ^{1}H resonance after the GSLAC with a ^{14}NV, using various τ times and with or without a π pulse to initialise the NV in $|-1\rangle$ or $|0\rangle$, respectively. The red solid lines are the global fit to the whole data set using Supplementary Eqs. (8), (9) and (11). (b) NMR spectra near the ^{1}H Larmor frequency obtained with the XY8 method using the same NV as in (a). The PL intensity after the XY8 sequence is measured as a function of the wait time τ between the π pulses, thus effectively scanning the probe frequency $\omega_\tau = \pi/\tau$. The external magnetic field has a strength $B = 300$ G, and the total number of π pulses is $N = 256$. The two curves differ by the final RF pulse of the XY8 sequence, a $\frac{\pi}{2}$ pulse to project onto $|-1\rangle$ or a $\frac{3\pi}{2}$ pulse to project onto $|0\rangle$.

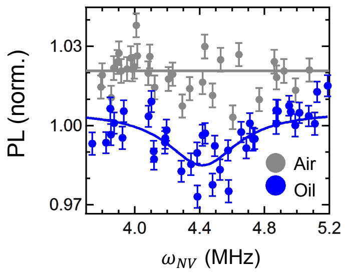

Supplementary Figure 4. **Control measurement to confirm source of proton signal.** Comparative oil (blue) and air (grey) measurement around the expected NV-proton resonance for the same ^{14}NV with a probe time of $\tau = 200$ μs. Both sets of data is fitted with lorentzians, limimting the transition to within 3 standard deviations of the expected NV-proton transition frequency.

Parameter	^{14}N	^{15}N
$D_{\mathrm{NV}}/2\pi$	2.87 GHz	2.87 GHz
$a_{\parallel}/2\pi$	-2.14 MHz	3.03 MHz
$a_{\perp}/2\pi$	-2.70 MHz	3.65 MHz
$\gamma_{\mathrm{NV}}/2\pi$	-28.035 GHz/T	-28.035 GHz/T
$\gamma_{\mathrm{N}}/2\pi$	3.077 MHz/T	-4.316 MHz/T
$q/2\pi$	-4.945 MHz	0 MHz

Supplementary Table 1. **Constants of the NV centre Hamiltonian.** Parameters of the NV centre Hamiltonian expressed in Supplementary Eq. 12. All constants are taken from Supplementary Ref. [4].

SUPPLEMENTARY NOTE 1. MODELLING OF T1-RELAXOMETRY MEASUREMENTS

Relaxation from a semi-infinite nuclear spin bath

Consider an NV interacting with a single (i^{th}) environmental proton, where the proton is in the correct initial state to cause decay of the initialised NV, i.e. $|\uparrow\rangle$ after the ground state level anti-crossing (GSLAC) or $|\downarrow\rangle$ before the GSLAC. The induced relaxation rate $\Gamma^i_{1,\text{ext}}$, when at the resonance point, is [2]

$$\Gamma^{i\pm}_{1,\text{ext}} = \frac{1}{2\Gamma_2^*}\left(\frac{\mu_0\gamma_{\text{NV}}\gamma_t\hbar}{4\pi}\right)^2\left(\frac{3\sin^2\theta_i - 1 \pm 1}{r_i^3}\right)^2 \tag{1}$$

where θ_i is the polar angle of the NV-proton separation relative to their shared quantisation axis, r_i is the separation distance, and $\Gamma_2^* = \Gamma_{2,\text{NV}}^* + \Gamma_{2,\text{H}}^* \approx \Gamma_{2,\text{NV}}^*$. There are two possible transitions of the nuclear spin. The $|\uparrow\rangle \to |\downarrow\rangle$ transition takes the "+" sign of the "±" while the $|\downarrow\rangle \to |\uparrow\rangle$ transition takes the "−" sign. These occur after and before the GSLAC respectively for a ^{1}H nuclear spin. The total extrinsic decay rate for an ensemble of hydrogen atoms is then just a linear sum over all the decay rates contributed by the individual protons,

$$\Gamma^{\pm}_{1,\text{ext}} = \sum_i \Gamma^{i\pm}_{1,\text{ext}} \tag{2}$$

$$\approx \frac{\rho}{4\Gamma_2^*}\left(\frac{\mu_0\gamma_{\text{NV}}\gamma_t\hbar}{4\pi}\right)^2\int_V\left(\frac{3\sin^2\theta - 1 \pm 1}{r^3}\right)^2 dV \tag{3}$$

where ρ is the density of protons within the sample and V is the volume occupied by the protons. There is a factor of $\frac{1}{2}$ included since only half of the protons are in the correct state resonant with the NV, while the other half does not interact with the NV. In order to complete the integral, we need to transform into the (x, y, z) basis of the (100) surface, where $\hat{z}$ is the outward surface normal. This means that the integral will be over a semi-infinite layer $z > d$ where d is the NV depth within the diamond. Applying these transformations and solving the integrals gives

$$\int_{z>d}\left(\frac{3\sin^2\theta}{r^3}\right)^2 dV = \frac{19\pi}{24d^3} \tag{4}$$

and

$$\int_{z>d}\left(\frac{3\sin^2\theta - 2}{r^3}\right)^2 dV = \frac{\pi}{8d^3}. \tag{5}$$

The extrinsic decay rate for the NV on resonance after (+) and before (-) the GSLAC is therefore

$$\Gamma^{+}_{1,\mathrm{ext}} = \frac{\rho}{4\Gamma_2^*}\left(\frac{\mu_0\gamma_{\mathrm{NV}}\gamma_t\hbar}{4\pi}\right)^2\frac{19\pi}{24d^3} \tag{6}$$

$$\Gamma^{-}_{1,\mathrm{ext}} = \frac{\rho}{4\Gamma_2^*}\left(\frac{\mu_0\gamma_{\mathrm{NV}}\gamma_t\hbar}{4\pi}\right)^2\frac{\pi}{8d^3}. \tag{7}$$

Photoluminescence response

We now link the relaxation rate from Supplementary Eqs. 6 and 7, to the PL signal measured from the NV. For near-surface NV centres, the relaxation time, T_1, is generally governed by magnetic noise from the surface rather than by phonons as for deep NVs. To analyse the spin dynamics of near-surface NV centres, we therefore consider the model of Supplementary Figure 1, where the three spin states of the NV electronic ground state, $|0,\pm1\rangle$, are linked via transitions rates k_+ and k_- associated with the transitions $|0\rangle \leftrightarrow |+1\rangle$ and $|0\rangle \leftrightarrow |-1\rangle$, respectively. The corresponding populations are denoted n_0, n_{+1} and n_{-1}, respectively. Those two transition rates depend on the magnetic field, which forms the basis for our method of T_1 spectroscopy.

Solving the rate equations yields the populations as a function of time, which gives for n_0,

$$\begin{aligned} n_0(\tau) = \tfrac{1}{3} &+ \frac{[3n_0(0)-1]\xi - [3n_{+1}(0)-1]k_+ - [3n_{-1}(0)-1]k_-}{6\xi}e^{-(\kappa+\xi)\tau} \\ &+ \frac{[3n_0(0)-1]\xi + [3n_{+1}(0)-1]k_+ + [3n_{-1}(0)-1]k_-}{6\xi}e^{-(\kappa-\xi)\tau}, \end{aligned} \tag{8}$$

where the initial populations satisfy the closed-system condition $n_0(0)+n_{+1}(0)+n_{-1}(0) = 1$, and we defined

$$\kappa = k_+ + k_-$$
$$\xi = \sqrt{k_+^2 + k_-^2 - k_+k_-}\ .$$

The PL intensity at the start of the readout laser pulse following a wait time τ, $I_{\mathrm{PL}}(\tau)$, can be expressed as

$$\begin{aligned} I_{\mathrm{PL}}(\tau) &= I_A n_0(\tau) + I_B[n_{+1}(\tau) + n_{-1}(\tau)] \\ &= I_B + (I_A - I_B)n_0(\tau) \end{aligned} \tag{9}$$

where I_A and $I_B < I_A$ are the PL rates associated with spin states $|0\rangle$ and $|\pm 1\rangle$. By inserting Supplementary Eq. (8) into Supplementary Eq. (9), we obtain an equation for the PL intensity as a function of τ, the rates $k_\pm$, the initial populations following the initialisation pulse, and the PL rates. We note that near the GSLAC ($B \approx 1024$ G), k_+ is governed by magnetic noise at high frequency (≈ 5.74 GHz) while k_- is sensitive to low-frequency magnetic noise ($\lesssim 10$ MHz). As a consequence, one generally has $k_- \gg k_+$, which results in a simple expression for $I_{\rm PL}(\tau)$,

$$I_{\rm PL}(\tau) \approx I_0 \left[1 + \mathcal{C} e^{-\Gamma_{1,\rm tot}\tau}\right], \tag{10}$$

where I_0 and $\mathcal{C}$ are constants, and we defined $\Gamma_{1,\rm tot} = 2k_-$. This corresponds to Eq. (2) of the main text. Near a cross-relaxation resonance, $\Gamma_{1,\rm tot}$ can be expressed as [2, 3]

$$\Gamma_{1,\rm tot}(B) = \Gamma_{1,\rm int} + \frac{\Gamma^{\pm}_{1,\rm ext}}{1 + \left(\frac{\omega_{\rm NV}(B) - \omega_{\rm t}(B)}{\Gamma_2^*}\right)^2}, \tag{11}$$

composed of the intrinsic decay ($\Gamma_{1,\rm int}$) and the extrinsic component from the interaction with a background target spin, with $\omega_{\rm NV}(B)$ the NV transition frequency, $\omega_{\rm t}(B)$ the transition frequency of the target spins, and $\Gamma^{\pm}_{1,\rm ext}$ the relaxation rate at the NV-target resonance as expressed in Supplementary Eqs. (6) and (7). By inserting Supplementary Eq. (11) into Eq. (10), one obtains an equation that can be fit to the spectrum $I_{\rm PL}(\tau, B)$ recorded at a given τ, using the values of I_0 and $\mathcal{C}$ obtained from a calibration measurement. From the fit, we infer the quantities $\Gamma_{1,\rm int}$, $\Gamma^{\pm}_{1,\rm ext}$ and Γ_2^*. From these, it is possible to deduce the NV depth, d, using Supplementary Eq. (6) or (7). Note that a generalised form of $I_{\rm PL}(\tau)$, which does not assume $k_- \gg k_+$, can be used instead of Supplementary Eq. (10), by using Supplementary Eq. (8). However, there are more constants to be determined (in particular, k_+), which requires additional calibration measurements. This was done in Supplementary Note 3, in order to precisely determine the NV depth.

SUPPLEMENTARY NOTE 2. RESONANCE FREQUENCIES

NV and nuclear spin resonance frequencies

The NV centre has an electronic spin-1 ground state triplet. The spin Hamiltonian can be written as

$$\begin{aligned}\frac{\mathcal{H}_{\mathrm{NV}}}{\hbar} =& D_{\mathrm{NV}} S_z^2 - \gamma_{\mathrm{NV}} B S_z + a_{\parallel} S_z I_z \\ &+ a_{\perp}\left(S_x I_x + S_y I_y\right) + q I_z^2 - \gamma_N B I_z. \end{aligned} \tag{12}$$

where $\mathbf{S}$ and $\mathbf{I}$ are the electron and nuclear spin operators where the nuclear spin is spin-1 for an NV composed of a ^{14}N and spin-1/2 for an NV composed of a ^{15}N (referred to hereafter by the notation ^{14}NV and ^{15}NV respectively). Additionally, D_{NV} is the NV zero-field splitting, $a_{\parallel}$ and $a_{\perp}$ are the NV centre's axial and non-axial hyperfine parameters, q is the nuclear quadrupole moment, γ_{NV} and γ_N are the NV electron and nuclear spin gyromagnetic ratios respectively and B is a magnetic field aligned with the NV centre's symmetry axis. These constants are defined in Supplementary Table **1**.

From this we can solve for the eigenvectors of both the ^{14}NV and ^{15}NV systems. A dipole-dipole interaction can cause the ^{14}NV to transition from its initialised $|0,+1\rangle$ state to $|-1,+1\rangle$. Here the notation $|m_S, m_I\rangle$ refers to the projections of the NV electron spin (m_S) and nuclear spin (m_I). The eigenstates containing a portion of the $|-1,+1\rangle$ state [1] have energy transitions from the initial state

$$\begin{aligned}\omega_{\mathrm{NV}} =& \frac{1}{2}\left(D_{\mathrm{NV}} - a_{\parallel} + \gamma_{\mathrm{NV}} B + \gamma_{\mathrm{N}} B - q\right) \\ &\pm \frac{1}{2}\sqrt{4a_{\perp}^2 + \left(a_{\parallel} - D_{\mathrm{NV}} - q - \gamma_{\mathrm{NV}} B + \gamma_{\mathrm{N}} B\right)^2}. \end{aligned} \tag{13}$$

Similarly, the ^{15}NV initialises into the $\left|0,+\frac{1}{2}\right\rangle$ state and a dipole-dipole interaction can cause a transition to the $\left|-1,+\frac{1}{2}\right\rangle$. The transitions to states containing the $\left|-1,+\frac{1}{2}\right\rangle$ state are

$$\begin{aligned}\omega_{\mathrm{NV}} =& \frac{1}{4}\left(2D_{\mathrm{NV}} - a_{\parallel} + 2\gamma_{\mathrm{NV}} B + 2\gamma_{\mathrm{N}} B\right) \\ &\pm \frac{1}{4}\sqrt{a_{\parallel}^2 - 4a_{\parallel}\left(D_{\mathrm{NV}} + \gamma_{\mathrm{NV}} B + \gamma_{\mathrm{N}} B\right) + 8a_{\perp}^2 + \left(D_{\mathrm{NV}} + \gamma_{\mathrm{NV}} B + \gamma_{\mathrm{N}} B\right)^2}. \end{aligned} \tag{14}$$

The target nuclear spin transition energies are $\omega_{\mathrm{t}} = \pm\gamma_{\mathrm{t}} B$ and thus we have resonances when $\omega_{\mathrm{t}} = \omega_{\mathrm{NV}}$. Using the constants defined in Table **1**, we find that the high-field NV-proton resonance point for a ^{14}NV is at B = 1025.8 G and the pair of resonance points for ^{15}NV are at B = 1022.1 G and 1024.1 G.

^{15}NV crossing feature

For the case of ^{15}NV there is also a feature where the $\left|0, +\frac{1}{2}\right\rangle$ and $\left|-1, -\frac{1}{2}\right\rangle$ states cross at 1024.1 G as discussed in Ref. [1]. Supplementary Figure 2a shows the ODMR for a ^{15}NV across the GSLAC. The signal around the expected NV-proton resonance is shown in Supplementary Figure 2b with a strong decay of $\approx$ 1 MHz. This is far stronger than expected for an NV interacting with an external proton bath and hence we conclude that this is due to the crossing feature rather than the NV-proton resonance. In order to separate these features, we used a ^{15}NV coupled strongly ($\approx$ 4 MHz) to an environmental ^{13}C with the ODMR of this NV shown in Supplementary Figure 2c.

The Hamiltonian of the coupled NV-^{13}C system can be written as

$$\mathcal{H}_{\text{Sys}} = \mathcal{H}_{\text{NV}} + \mathcal{H}_{\text{C}} + \mathcal{H}_{\text{Hyp}} \tag{15}$$

where $\mathcal{H}_{\text{C}}$ is the Hamiltonian of the ^{13}C nuclear spin and $\mathcal{H}_{\text{Hyp}}$ is the Hamiltonian of the hyperfine interaction between the ^{15}NV and the ^{13}C. These are

$$\frac{\mathcal{H}_{\text{C}}}{\hbar} = -\gamma_{\text{C}} B I_z \tag{16}$$

$$\frac{\mathcal{H}_{\text{Hyp}}}{\hbar} = A_{\parallel}^{C}\left(S_z I_z\right) + A_{\perp}^{C}\left(S_x I_x + S_y I_y\right) \tag{17}$$

where $\gamma_{\text{C}}/2\pi = 10.705\,\text{MHz/T}$ is the gyromagnetic ratio of ^{13}C, $A_{\parallel}^{C}$ and $A_{\perp}^{C}$ are the axial and transverse components of the NV-^{13}C hyperfine and $\hat{S}$ and $\hat{I}$ are the ^{15}NV and ^{13}C spin operators respectively. Once again this Hamiltonian could be diagonalised to solve the transition frequencies.

There were however the two unknowns of the NV-^{13}C hyperfine parameters. Comparing the splitting in our ODMR spectrum shown in Supplementary Figure 2c with those hyperfine parameters quoted in Ref. [5] we take $A_{\parallel}^{C}$ to be 4.12 MHz. The transverse component, $A_{\perp}^{C}$ is found from fitting our measured energy transitions giving $A_{\perp}^{C} = 3.0 \pm 0.1\,\text{MHz}$.

SUPPLEMENTARY NOTE 3. COMPARISON BETWEEN T_1 AND XY8 NMR

Theoretical sensitivity

Here we compare the sensitivity of T_1 spectroscopy for nuclear spin detection to the current standard of T_2-based spectroscopy, which relies on locking a dynamical decoupling pulse sequence (usually XY8-N) to the nuclear spin's Larmor frequency [6, 7]. We do this via a comparison of the signal-to-noise ratio (SNR) derived under identical conditions, in the case of a shallow NV centre detecting an ensemble of nuclear spins as in the geometry of main text Supplementary Figure 1a.

For T_1 spectroscopy, the measurement sequence consists of a 3-μs laser pulse followed by a wait time τ assumed to be much longer than 3 μs. The intensity $I_{\rm PL}(\tau)$ is obtained by counting the photons within a read-out time $t_{\rm ro} = 300$ ns. As a result, the PL signal is acquired only for a fraction $t_{\rm ro}/\tau$ of the total experiment time, $T_{\rm tot}$. The total number of photons detected can be expressed as (see Supplementary Eq. (10))

$$\mathcal{N}(\Gamma_{1,\rm ext},\tau) = \mathcal{R}T_{\rm tot}\frac{t_{\rm ro}}{\tau}\left[1-\mathcal{C}+\mathcal{C}e^{-(\Gamma_{1,\rm int}+\Gamma_{1,\rm ext})\tau}\right] \tag{18}$$

where $\mathcal{R}$ is the photon count rate under continuous laser excitation, $\Gamma_{1,\rm int}$ is the intrinsic longitudinal relaxation rate of the NV, $\Gamma_{1,\rm ext}$ is the extrinsic relaxation rate of the NV induced by the on-resonance target spins, and $\mathcal{C}$ is the contrast between $|0\rangle$ and a mixture of $|0\rangle$ and $|-1\rangle$ (assuming $k_- \gg k_+$). The change in the number of photons caused by the presence of $\Gamma_{1,\rm ext}$ is

$$\begin{aligned}\Delta\mathcal{N}_{\rm signal}(\tau) &= \mathcal{N}(0,\tau)-\mathcal{N}(\Gamma_{1,\rm ext},\tau)\\ &= \frac{\mathcal{R}T_{\rm tot}t_{\rm ro}\mathcal{C}}{\tau}e^{-\Gamma_{1,\rm int}\tau}\left(1-e^{-\Gamma_{1,\rm ext}\tau}\right).\end{aligned} \tag{19}$$

The photon shot noise associated with the measurement is

$$\begin{aligned}\Delta\mathcal{N}_{\rm noise}(\tau) &= \sqrt{\mathcal{N}(\Gamma_{1,\rm ext},\tau)}\\ &\approx \sqrt{\frac{\mathcal{R}T_{\rm tot}t_{\rm ro}}{\tau}}\end{aligned} \tag{20}$$

where we used the approximation $\mathcal{C} \ll 1$. In the small signal regime ($\Gamma_{1,\rm ext} \ll \Gamma_{1,\rm int}$), the SNR is then

$$\begin{aligned}{\rm SNR}_{T_1}(\tau) &= \frac{\Delta\mathcal{N}_{\rm signal}(\tau)}{\Delta\mathcal{N}_{\rm noise}(\tau)}\\ &\approx \sqrt{\mathcal{R}T_{\rm tot}t_{\rm ro}}\mathcal{C}e^{-\Gamma_{1,\rm int}\tau}\Gamma_{1,\rm ext}\sqrt{\tau}.\end{aligned} \tag{21}$$

The SNR is maximised for a probe time $\tau = 1/2\Gamma_{1,\mathrm{int}}$ and becomes

$$\mathrm{SNR}_{T_1,\mathrm{max}} \approx \sqrt{\mathcal{R}T_{\mathrm{tot}}t_{\mathrm{ro}}}\mathcal{C}e^{-1/2}\Gamma_{1,\mathrm{ext}}\sqrt{\frac{1}{2\Gamma_{1,\mathrm{int}}}}. \tag{22}$$

Similarly, we can write the number of photons detected in a T_2-based sensing scheme as

$$\mathcal{N}(\Gamma_{2,\mathrm{ext}},\tau) = \mathcal{R}T_{\mathrm{tot}}\frac{t_{\mathrm{ro}}}{\tau}\left[1-\mathcal{C}+\mathcal{C}e^{-(\Gamma_{2,\mathrm{int}}\tau)^2}e^{-(\Gamma_{2,\mathrm{ext}}\tau)^2}\right] \tag{23}$$

where $\Gamma_{2,\mathrm{int}}$ is the intrinsic transverse relaxation rate in the absence of signal (under the considered dynamical decoupling sequence, e.g. XY8-N), $\Gamma_{2,\mathrm{ext}}$ is the additional transverse relaxation rate induced by the target spins, and all other constants are defined as before. Note that the time dependence is now Gaussian rather than exponential [7]. In the small signal regime ($\Gamma_{2,\mathrm{ext}} \ll \Gamma_{2,\mathrm{int}}$), the SNR is maximised for a probe time $\tau = \sqrt{3}/2\Gamma_{2,\mathrm{int}}$ and is

$$\mathrm{SNR}_{T_2,\mathrm{max}} \approx \sqrt{\mathcal{R}T_{\mathrm{tot}}t_{\mathrm{ro}}}\mathcal{C}e^{-3/4}(\Gamma_{2,\mathrm{ext}})^2\left(\frac{\sqrt{3}}{2\Gamma_{2,\mathrm{int}}}\right)^{3/2}. \tag{24}$$

We now consider a particular sensing situation which allows us to express the signal as measured in T_1 or T_2 spectroscopy. Namely, we look at the case of a single NV centre located near the diamond (100) surface, detecting a semi-infinite volume of nuclear spins (see main text Fig. 1a). The induced longitudinal relaxation rate for the resonance past the GSLAC is given by Supplementary Eq. (6), that is,

$$\Gamma_{1,\mathrm{ext}} = \frac{\rho}{4\Gamma_2^*}\left(\frac{\mu_0\gamma_{\mathrm{NV}}\gamma_t\hbar}{4\pi}\right)^2\frac{19\pi}{24d^3} \tag{25}$$

where d is the NV depth, ρ is the density of target nuclear spins, γ_t is the gyromagnetic ratio of the target spins. In the T_2-based locking technique, the induced decoherence rate is [7]

$$(\Gamma_{2,\mathrm{ext}})^2 = \rho\left(\frac{\mu_0\gamma_{\mathrm{NV}}\gamma_t\hbar}{4\pi}\right)^2\frac{5}{48\pi d^3}. \tag{26}$$

We can thus write the SNR in both sensing schemes as

$$\mathrm{SNR}_{T_1,\mathrm{max}} \approx \mathcal{A}\times\frac{19\pi e^{-1/2}}{96\sqrt{2}}\times\frac{1}{\Gamma_2^*\sqrt{\Gamma_{1,\mathrm{int}}}} \tag{27}$$

$$\mathrm{SNR}_{T_2,\mathrm{max}} \approx \mathcal{A}\times\frac{5(3/e)^{3/4}}{48\pi 2^{3/2}}\times\left(\frac{1}{\Gamma_{2,\mathrm{int}}}\right)^{3/2} \tag{28}$$

where $\mathcal{A}$ is a shared constant. Evaluating the numeric factors and using the conventional notations $T_2^* = 1/\Gamma_2^*$, $T_1 = 1/\Gamma_{1,\mathrm{int}}$ and $T_2 = 1/\Gamma_{2,\mathrm{int}}$, we obtain the ratio between the SNRs,

$$\frac{\mathrm{SNR}_{T_1,\mathrm{max}}}{\mathrm{SNR}_{T_2,\mathrm{max}}} \approx 21.1\times\frac{T_2^*\sqrt{T_1}}{(T_2)^{3/2}}. \tag{29}$$

Comparison of the extracted depth

Here we detail the procedure to infer the NV depth from the experimental data, for both T_1 and XY8 methods using the same NV centre. For the T_1 method, the full data set is shown in Supplementary Figure 3a. The PL intensity at the start of the readout laser pulse (normalised by that at the end of the pulse) is measured as a function of the NV transition frequency, $\omega_{\rm NV}$, varied by sweeping the magnetic field and determined by recording an ODMR spectrum. Spectra are recorded with different wait times, $\tau = 1,\ 50,\ 100\ \mu$s, either all-optically or following an RF π pulse to initialise the NV in the $|-1\rangle$ state. Although only one of those spectra is needed to detect the ^{1}H signal (e.g., main text Fig. 4a shows the all-optical $\tau = 100\ \mu$s spectrum), the use of several evolution times and a different initial NV state via the π pulses enables us to fit the data using the general form for the spin population decay, as given in Supplementary Eq. (8). We thus fit the data to Supplementary Eq. (9) together with Supplementary Eq. (8), which gives $k_+ = 164 \pm 10$ s^{-1}, $k_{-,\rm int} = \Gamma_{1,\rm int}/2 = 2285 \pm 10$ s^{-1}, $\Gamma^+_{1,\rm ext} = 11.9 \pm 0.1 \times 10^3$ s^{-1}, $\Gamma_2^* = 637 \pm 5 \times 10^3$ s^{-1} and $\omega_{\rm t}/2\pi = 4.40 \pm 0.01$ MHz. The NV depth is then deduced using Supplementary Eq. (6) together with the gyromagnetic ratio of hydrogen, $\gamma_t = 2.67513 \times 10^8$ s^{-1}T^{-1}, and the proton density of PMMA, $\rho = 56$ nm^{-3}, yielding $d = 10.7 \pm 0.1$ nm.

For the XY8 method, we followed the procedure of Ref. [7]. Supplementary Figure 3b shows the raw signal obtained with the same NV as before, with $N = 256$ π pulses under a magnetic field $B = 300$ G. The two curves, I_+ and I_-, are combined to give the normalised contrast $S = \frac{I_+ - I_-}{I_+ + I_-}$ plotted in main text Fig. 4b. This is then fit to Eqs. (1) and (4) of Ref. [7], which includes a finite dephasing time of the target spins, denoted T^*_{2n}. The fit to the data yields $T^*_{2n} = 16.3 \pm 0.7\ \mu$s, a Larmor frequency $\omega_{\rm H}/2\pi = 1.285 \pm 0.001$ MHz, and a depth $d = 10.5 \pm 0.1$ nm, in excellent agreement with the depth derived via the T_1 method.

SUPPLEMENTARY NOTE 4. CONTROL MEASUREMENT

In order to confirm the source of the decay as hydrogen nuclear spins, we conducted a comparative oil and air measurement with a ^{14}NV. A control measurement was taken with the NV surface exposed to air with a probe time $\tau = 200\ \mu$s. The data is shown in grey in Supplementary Figure 4a. A best-fit Lorentzian with a central transition limited to

within experimental error ($\approx 2\%$) of the theoretical transition frequency shows no decay. In comparison, the oil measurement on the same NV (shown in blue) shows a contrast of $\approx 1.7\%$ at 4.42 ± 0.06 MHz, within errors of the theoretical transition.

SUPPLEMENTARY REFERENCES

[1] D. A. Broadway, J. D. A. Wood, L. T. Hall, A. Stacey, M. Markham, D. A. Simpson, J-P. Tetienne, and L. C. L. Hollenberg, Anticrossing Spin Dynamics of Diamond Nitrogen-Vacancy Centers and All-Optical Low-Frequency Magnetometry, Phys. Rev. Appl. 6, 064001 (2016).

[2] J. D. A. Wood, D. A. Broadway, L. T. Hall, A. Stacey, D. A. Simpson, J-P. Tetienne, and L. C. L. Hollenberg, Wide-band, nanoscale magnetic resonance spectroscopy using quantum relaxation of a single spin in diamond, Phys. Rev. B 94, 155402 (2016).

[3] L. T. Hall, P. Kehayias, D. A. Simpson, A. Jarmola, A. Stacey, D. Budker, and L. C. L. Hollenberg, Detection of nanoscale electron spin resonance spectra demonstrated using nitrogen-vacancy centre probes in diamond, Nat. Commun. 7, 10211 (2016).

[4] M. W. Doherty, et al., The nitrogen-vacancy colour centre in diamond, Phys Rep 528, 1 (2013).

[5] A. Dréau, J. R. Maze, M. Lesik, J.-F. Roch, V. Jacques, High-resolution spectroscopy of single NV defects coupled with nearby ^{13}C nuclear spins in diamond, Phys. Rev. B 85, 134107 (2012).

[6] T. Staudacher, F. Shi, S. Pezzagna, J. Meijer, J. Du, C. A. Meriles, F. Reinhard, and J. Wrachtrup, Nuclear magnetic resonance spectroscopy on a (5-nanometer)^3 sample volume, Science 339, 561-563 (2013).

[7] L. M Pham et al., NMR technique for determining the depth of shallow nitrogen-vacancy centers in diamond, Phys. Rev. B 93, 045425 (2015).
